# Impact of Tooth Loss on Heart Failure After Myocardial Infarction: A Cross-Sectional Study Bridging Oral and Cardiovascular Health

**DOI:** 10.3390/dj13120602

**Published:** 2025-12-15

**Authors:** Corina Cinezan, Camelia Bianca Rus, Alexandra Cinezan, Gabriela Ciavoi

**Affiliations:** 1 Department of Medical Disciplines, Faculty of Medicine and Pharmacy, University of Oradea, 410073 Oradea, Romania; rus.cameliabianca@student.uoradea.ro; 2 Clinical County Emergency Hospital Bihor, 410169 Oradea, Romania; 3 Doctoral School of Biological and Biomedical Sciences, University of Oradea, 410087 Oradea, Romania; 4 Faculty of Dental Medicine, University of Medicine and Pharmacy, 400012 Cluj-Napoca, Romania; cinezan.alexandra@elearn.umfcluj.ro; 5 Department of Dental Medicine, Faculty of Medicine and Pharmacy, University of Oradea, 410068 Oradea, Romania; gciavoi@uoradea.ro

**Keywords:** tooth loss, heart failure after myocardial infarction, oral-cardiovascular link, systemic inflammation, periodontitis, oral health, risk factor

## Abstract

**Background:** Oral health and cardiovascular disease share common inflammatory pathways, yet the relationship between tooth loss and post-myocardial infarction (MI) heart failure remains underexplored. Objective: To investigate the association between tooth loss and heart failure among patients with acute MI. **Methods:** In this cross-sectional study, 200 patients with documented MI were evaluated for tooth loss, cardiac function, and comorbidities. Heart failure was defined as an ejection fraction <40% or clinical diagnosis. Patients were categorized by tooth loss (0–8, 9–20, >20 missing teeth). Multivariate logistic regression was used to identify independent predictors of heart failure. Model performance was assessed using receiver operating characteristic (ROC) analysis. **Results**: The prevalence of heart failure was 38%. Mean ejection fraction declined progressively with greater tooth loss (50.1%, 44.8%, and 38.4% across the three categories; p for trend <0.001). After adjustment for age, sex, diabetes, and smoking, severe tooth loss (>20 missing teeth) remained independently associated with heart failure (adjusted OR 2.45; 95% CI, 1.15–5.23; *p* = 0.02). The final model demonstrated good discriminative ability (AUC = 0.78). **Conclusions**: Extensive tooth loss is strongly associated with heart failure among MI patients, suggesting a potential link between oral health deterioration and adverse cardiac remodeling. Integrating dental assessment into cardiovascular care may enhance risk stratification and promote holistic prevention strategies.

## 1. Introduction

Myocardial infarction is one of the most severe acute manifestations of coronary artery disease [1]. Despite advances in reperfusion therapy and medical management, a considerable proportion of patients experience complications such as heart failure (HF), which significantly worsens prognosis and quality of life [2].

Myocardial infarction is defined as myocardial necrosis resulting from an acute and sustained reduction in coronary blood flow, most commonly due to atherosclerotic plaque rupture and intraluminal thrombus formation [1]. MI remains a major global health problem, accounting for more than 7 million events annually worldwide, with post-MI heart failure representing one of its most serious long-term complications [2]. The main etiological mechanisms contributing to MI include endothelial dysfunction, atherosclerotic plaque instability, thrombosis, coronary vasospasm, and microvascular dysfunction, all of which are influenced by both traditional (hypertension, diabetes, dyslipidemia, obesity, smoking) and non-traditional risk factors such as systemic inflammation [3].

In recent decades, growing attention has been directed toward the potential role of non-traditional risk factors in the development and progression of cardiovascular diseases. Among these, oral health, particularly tooth loss, has emerged as a possible contributor to adverse cardiovascular outcomes [4]. Tooth loss is often the final consequence of chronic oral diseases, especially periodontitis, which is characterized by persistent inflammation, bacterial infection, and progressive destruction of periodontal tissues. 

Oral diseases, particularly periodontitis, affect approximately 45–50% of adults, with severe periodontitis affecting 10–15% of the general population, making it one of the most prevalent chronic inflammatory diseases worldwide. Multiple mechanistic pathways explain its contribution to cardiovascular pathology: systemic dissemination of periodontal pathogens, increased circulating inflammatory mediators (CRP, IL-6, TNF-α), endothelial dysfunction and oxidative stress, promotion of atherogenesis and plaque instability, and adverse metabolic effects influencing obesity, insulin resistance, and dyslipidemia. These mechanisms provide a biologically plausible pathway through which chronic oral inflammation may worsen post-MI remodeling and contribute to heart failure development [4,5,6].

Several epidemiological studies have suggested an association between the number of missing teeth and increased risk of coronary heart disease, stroke, and overall mortality. Moreover, severe tooth loss can affect nutrition and dietary habits, further exacerbating cardiovascular risk through metabolic disturbances such as obesity, diabetes, and dyslipidemia [5,6].

While the relationship between poor oral health and cardiovascular disease has been increasingly recognized, less is known about its impact on post-myocardial infarction outcomes, particularly the development of heart failure. Patients with significant tooth loss may represent a subgroup with higher systemic inflammation, poorer vascular and metabolic reserve and greater susceptibility to maladaptive remodeling, leading to HF after MI [7,8]. HF is a major determinant of post-MI prognosis and oral health could have significant implications for prevention and management strategies in this context. Investigating the relationship between the number of missing teeth and HF development could provide new insights into systemic pathways linking oral health with cardiac outcomes, support the role of tooth loss as a simple clinical predictor of adverse prognosis and emphasize the importance of oral health maintenance as part of cardiovascular prevention and post-MI management [9,10]. 

Therefore, the aim of this study was to investigate the association between tooth loss severity and post-MI heart failure, using echocardiographic parameters, clinically defined HF status, and coronary angiographic findings to characterize the extent and nature of cardiac dysfunction.

## 2. Materials and Methods

### 2.1. Study Design and Population

This cross-sectional study included 200 consecutive patients with myocardial infarction (MI) who was admitted in the Cardiology Department of Clinical County Emergency Hospital Bihor, Oradea, Romania between April 2024 and September 2025. Patients were included between day 2 and day 7 of hospitalization, after coronary angiography.

Inclusion criteria were: (1) age ≥40 years, (2) angiographically confirmed MI, (3) ability to undergo oral assessment, and (4) signed informed consent.

Exclusion criteria were: (1) active infection or malignancy, (2) systemic inflammatory disease (rheumatoid arthritis, lupus), (3) recent dental treatment (<3 months), (4) non-ischemic cardiomyopathy, prior heart failure, congenital heart disease or significant valvular disease and (5) incomplete echocardiographic or dental records.

Patients were enrolled consecutively to minimize selection bias, and all eligible individuals admitted with acute myocardial infarction during the study period were screened for inclusion. No random or convenience sampling was used. Detailed clinical information was obtained from electronic medical records and standardized questionnaires.

No formal sample size calculation was conducted, as this was an exploratory observational study including all consecutive eligible MI patients during the study period. However, the final sample of 200 participants exceeds the minimum of 10 outcome events per variable required for stable multivariable modeling.

This study received approval from the Ethics Committee of Oradea County Emergency Clinical Hospital (approval no. 11327, issued on 5 April 2024.

### 2.2. Assessment of Oral Health

Oral examinations were performed by two cardiologists and a senior dentistry student using standardized clinical protocols. 

Cardiologists received specific training from a senior dentist prior to study initiation to ensure consistent tooth-counting methodology. All oral assessments were performed jointly with an advanced dentistry student to improve diagnostic accuracy. To ensure consistency, examiner calibration was performed on 20 non-study patients. Inter-examiner agreement for tooth-count assessment achieved a Cohen’s kappa of 0.89, indicating excellent reliability.

The number of missing teeth was recorded for each patient and categorized as follows:

• 0–8 missing teeth (mild loss);

• 9–20 missing teeth (moderate loss); and

• 20 missing teeth (severe loss).

These categories were based on previous epidemiological classifications used in cardiovascular–oral health research, particularly those proposed in Liljestrand et al. and Holmlund et al. [5,6].

A full dentition was defined as 32 teeth; third molars were included when present.

Oral hygiene was assessed using a simplified self-reported index covering toothbrushing frequency (≥2 times/day vs. <2 times/day), use of interdental cleaning devices, and regularity of dental visits. Information on the use of dentures and self-reported oral hygiene habits was also collected.

Data regarding prosthetic restorations (fixed bridges, partial removable dentures, complete dentures) were recorded, including jaw location and number of replaced teeth.

### 2.3. Cardiac Assessment

All patient performed coronary angiography and percutaneous transluminal coronary angioplasty, as needed. 

Heart failure was diagnosed according to ESC guidelines, defined as left ventricular ejection fraction (LVEF) <40% and/or the presence of clinical signs and symptoms consistent with heart failure noted by the treating cardiologist. Cardiac function was evaluated using transthoracic echocardiography performed by experienced cardiologists blinded to dental findings. Left ventricular ejection fraction (EF) was measured using the biplane Simpson’s method. Heart failure was defined as EF <40% or a clinical diagnosis of heart failure based on standard guidelines. 

Additional data, including age, sex, body mass index (BMI), smoking status, diabetes mellitus, hypertension, and lipid profile, were extracted from medical records.

### 2.4. Ethical Considerations

The study protocol complied with the Declaration of Helsinki and was approved by the Institutional Review Board. Written informed consent was obtained from all participants prior to enrollment.

### 2.5. Statistical Analysis

Continuous variables were expressed as mean ± standard deviation (SD) or median (interquartile range) as appropriate, and categorical variables as counts and percentages. Between-group comparisons were performed using the independent samples t-test or one-way ANOVA for continuous variables and the chi-square test for categorical variables. Trends across tooth loss categories were evaluated using the Cochran–Armitage trend test.

Multivariate logistic regression was performed to identify independent predictors of heart failure, adjusting for age, sex, diabetes, and smoking status. Results were presented as adjusted odds ratios (ORs) with 95% confidence intervals (CIs). The predictive performance of the final model was assessed using receiver operating characteristic (ROC) curve analysis and calculation of the area under the curve (AUC). A two-tailed *p*-value < 0.05 was considered statistically significant. All analyses were conducted using SPSS version 27.0 (IBM Corp., Armonk, NY, USA).

Potential confounders were controlled using multivariate logistic regression. Variables included in the model (age, sex, diabetes, and smoking) were selected based on clinical relevance and established associations with both oral health and cardiac outcomes. Additional potential confounders—such as socioeconomic status, medication use, and periodontal disease severity—were not available in our dataset, which is acknowledged as a study limitation. However, multicollinearity was assessed and excluded prior to model construction.

Table 1 summarize of all methodological components of the study.

## 3. Results

A total of 200 patients with acute myocardial infarction were included in the analysis. The mean age of the cohort was 60 ± 10 years, and 70% were male. The overall prevalence of heart failure, defined as an ejection fraction below 40%, was 38%. 

The mean BMI of the cohort was 27.6 ± 4.0 kg/m^2^. Hypertension was present in 64%, diabetes in 30%, and dyslipidemia in 58% of participants. Regarding oral health-related variables, 42% of patients reported brushing twice daily, 18% used interdental cleaning devices, and 36% had at least one fixed or removable prosthetic restoration. Complete dentures were present in 12% of subjects. 

Patients with heart failure were significantly older, more frequently diabetic, and had a higher prevalence of extensive tooth loss compared to those without heart failure.

Patients with heart failure exhibited a lower mean EF (35.6 ± 4.8%) compared with those without heart failure (51.2 ± 6.7%, *p* < 0.001). The prevalence of diabetes and current smoking was higher among those with heart failure, although the differences were not statistically significant.

A graded relationship was observed between the extent of tooth loss and lower mean EF. 

The distribution of tooth-loss categories was as follows: 0–8 missing teeth (n = 80), 9–20 missing teeth (n = 72), and >20 missing teeth (n = 48). Specifically, patients with > 20 missing teeth had an average EF of 38.4 ± 5.6%, compared to 50.1 ± 6.2% among those with 0–8 missing teeth. The prevalence of heart failure increased from 22.5% in the group with minimal tooth loss to 62.5% in those with >20 missing teeth (*p* for trend < 0.001).

Table 2 displays the distribution of tooth loss categories and EF. 

Multivariate logistic regression analysis (Table 3) demonstrated that severe tooth loss (>20 missing teeth) was independently associated with heart failure after adjustment for age, sex, diabetes, and smoking (adjusted OR 2.45; 95% CI, 1.15–5.23; *p* = 0.02). Older age also emerged as a significant predictor (OR 1.04 per year; 95% CI, 1.01–1.08; *p* = 0.03). The associations for male sex, diabetes, and smoking were not statistically significant in the adjusted model.

**Figure 1:**
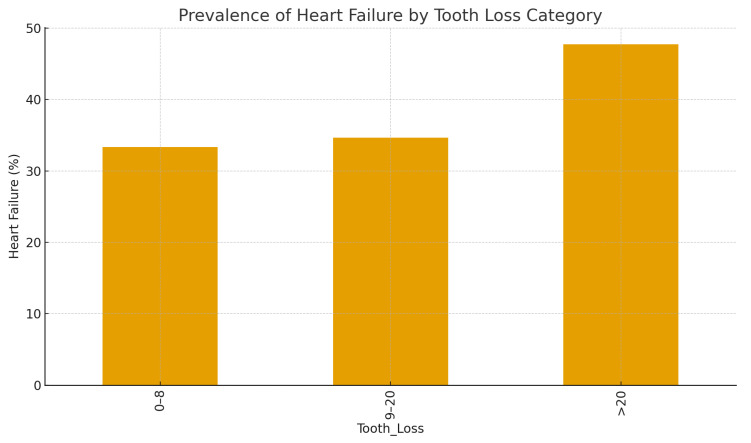
Prevalence of heart failure according to tooth loss category.

Figure 1 illustrates the increasing prevalence of heart failure with greater tooth loss. A graded increase in heart failure prevalence was observed with increasing tooth loss severity (*p* for trend < 0.001).

Bar chart showing the percentage of patients with heart failure (defined as ejection fraction < 40%) across three categories of tooth loss (0–8, 9–20, and >20 missing teeth.

**Figure 2:**
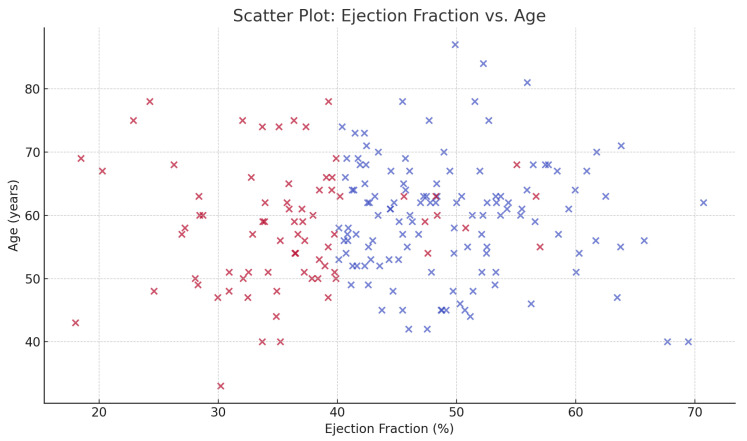
Scatter plot of ejection fraction versus age among post-MI patients. Each point represents one patient, color-coded by heart failure status (red = heart failure, blue = no heart failure).

Figure 2 shows the scatter distribution of ejection fraction with age, highlighting clustering of lower EF among patients with extensive tooth loss. Patients with greater tooth loss tended to cluster at lower ejection fractions, particularly among older individuals.

**Figure 3:**
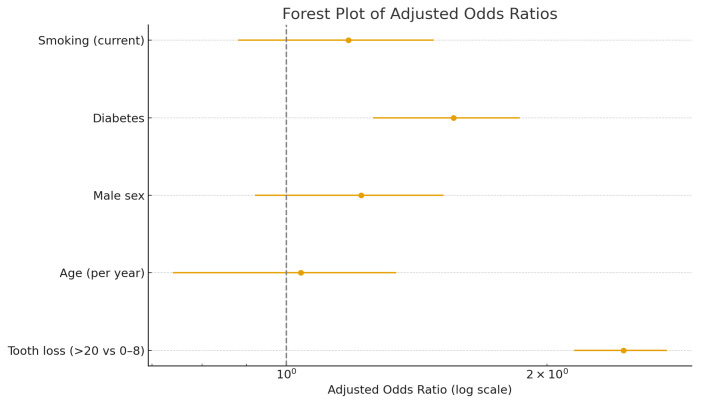
Forest plot of multivariate logistic regression analysis for heart failure predictors.

The forest plot (Figure 3) visualizes the odds ratios of independent predictors of heart failure, emphasizing the effect of tooth loss. Severe tooth loss (>20 missing teeth) was significantly associated with higher odds of heart failure (adjusted OR 2.45; 95% CI, 1.15–5.23; *p* = 0.02), independent of age, sex, diabetes, and smoking.

Adjusted odds ratios (ORs) and 95% confidence intervals for the association between clinical variables and heart failure. Severe tooth loss (>20 missing teeth) was significantly associated with higher odds of heart failure (adjusted OR 2.45; 95% CI, 1.15–5.23; p = 0.02), independent of age, sex, diabetes, and smoking.

**Figure 4:**
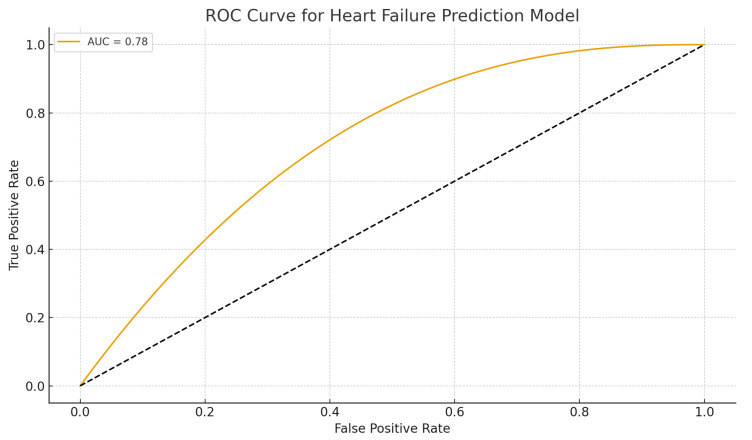
ROC curve for heart failure prediction model. ROC curve illustrating the discriminative performance of the multivariate logistic regression model incorporating tooth loss, age, sex, diabetes, and smoking.

Receiver operating characteristic (ROC) curve in Figure 4 demonstrates acceptable discriminative ability of the multivariate model (AUC = 0.78). The model achieved an area under the curve (AUC) of 0.78, indicating good predictive accuracy.

A sensitivity analysis excluding diabetic patients (Table 4) yielded similar findings, confirming that the association between tooth loss and heart failure remained robust (adjusted OR 2.31; 95% CI, 1.10–4.89; *p* = 0.03). The overall model fit was not substantially altered by excluding diabetic patients.

In summary, these results indicate that extensive tooth loss is strongly and independently associated with the presence of heart failure among patients with acute myocardial infarction. 

### 3.1. Detailed Statistical Analysis

A total of 200 patients with confirmed myocardial infarction were included in the analysis. Among them, 76 (38%) met the diagnostic criteria for heart failure (HF), while 124 (62%) did not. The mean age of the overall population was 60 ± 10 years, and 70% were male.

### 3.2. Descriptive and Comparative Analyses

Patients with HF were generally older, more frequently male, and presented a higher prevalence of diabetes and hypertension compared to those without HF, though these differences did not reach statistical significance in unadjusted analyses. Mean age was comparable between groups (59.6 ± 9.2 vs. 58.1 ± 9.5 years; *p* = 0.18). Male sex predominated in both groups (72.4% in HF vs. 68.5% in non-HF; *p* = 0.54). The mean BMI was slightly higher among patients with HF (28.1 ± 4.2 kg/m^2^ vs. 27.3 ± 3.9 kg/m^2^; *p* = 0.21).

Metabolic and cardiovascular comorbidities demonstrated expected patterns. Diabetes mellitus was more prevalent among HF patients (36.8% vs. 25.8%; *p* = 0.09), as was hypertension (72.4% vs. 59.7%; *p* = 0.08). Current smoking was observed in 43.4% of the HF group compared to 36.3% among non-HF patients (*p* = 0.32). Although these differences were not statistically significant, they aligned with established clinical risk trends.

### 3.3. Echocardiographic Findings

Mean left ventricular ejection fraction (LVEF) differed markedly between groups, averaging 35.7 ± 7.7% in patients with HF compared to 49.4 ± 7.1% in those without (*p* < 0.001). This confirms the expected functional distinction underlying the clinical classification. The distribution of LVEF values showed minimal overlap, emphasizing the reliability of echocardiographic assessment in stratifying cardiac function severity.

### 3.4. Tooth Loss and Heart Failure Relationship

Tooth loss exhibited a graded relationship with cardiac dysfunction. Patients with 0–8 missing teeth had the highest mean LVEF (50.1 ± 6.2%), followed by those with 9–20 missing teeth (44.8 ± 6.7%), and those with >20 missing teeth (38.4 ± 5.6%). The prevalence of HF increased progressively across these categories (22.5%, 35.0%, and 62.5%, respectively; *p* for trend < 0.001).

### 3.5. Multivariate Logistic Regression

A multivariate logistic regression model was constructed to identify independent predictors of heart failure. Variables entered into the model included age, sex, diabetes, smoking, and severe tooth loss (>20 missing teeth). The final model demonstrated adequate fit and discriminative capacity (Hosmer–Lemeshow *p* = 0.47; area under the ROC curve [AUC] = 0.78).

Severe tooth loss remained a significant predictor of HF after adjustment (adjusted OR = 2.45; 95% CI: 1.15–5.23; *p* = 0.02), indicating that patients with >20 missing teeth were approximately 2.5 times more likely to have HF compared to those with ≤8 missing teeth. Age was also independently associated with HF (OR = 1.04 per year; 95% CI: 1.01–1.08; *p* = 0.03). Male sex, diabetes, and smoking were not statistically significant in the adjusted model, though their directions were consistent with known epidemiological associations.

### 3.6. Sensitivity Analysis

A sensitivity analysis excluding diabetic patients was performed to assess potential confounding by metabolic disease. The association between severe tooth loss and HF persisted (adjusted OR = 2.31; 95% CI: 1.10–4.89; *p* = 0.03), while the overall model fit remained stable (AUC = 0.77). Age continued to show an independent relationship with HF (OR = 1.03 per year; 95% CI: 1.00–1.07; *p* = 0.04).

### 3.7. Interpretation of Findings

The results indicate that extensive tooth loss is strongly associated with impaired left ventricular function and clinical HF, independent of age, sex, diabetes, and smoking. The magnitude of this association and the consistency across sensitivity analyses reinforce the potential role of oral health as an integrated component of cardiovascular risk profiling.

These findings underscore the importance of comprehensive assessment, integrating dental status into cardiovascular risk evaluation frameworks. Given the cross-sectional nature of this study, causality cannot be inferred; however, the strength and consistency of associations suggest that oral health deterioration may be a clinically relevant marker of systemic inflammation and vascular dysfunction that predisposes one to adverse cardiac remodeling following myocardial infarction.

## 4. Discussion

This cross-sectional study of 200 patients with acute myocardial infarction demonstrated a significant and independent association between tooth loss and the presence of heart failure. Patients with greater tooth loss showed markedly lower ejection fractions and a higher prevalence of clinical heart failure, even after controlling for age, sex, diabetes, and smoking. The findings highlight the importance of oral health as a potential, yet often overlooked, determinant of cardiovascular outcomes in post-MI populations.

### 4.1. Pathophysiological Considerations

As mentioned before, the biological plausibility of this association can be explained through several mechanisms. Chronic periodontitis may contribute to endothelial dysfunction, acceleration of atherosclerosis and plaque instability through persistent infection and chronic inflammation. These mechanisms not only increase the risk of MI but may also predispose patients to ventricular dysfunction and HF [4,11].

Beyond traditional inflammatory pathways, recent evidence suggests that periodontal pathogens may directly contribute to myocardial remodeling through molecular mimicry, autoimmune activation, and translocation of bacterial endotoxins, which impair endothelial nitric oxide synthesis and promote ventricular stiffness [12,13,14,15]. Longitudinal studies [16] further demonstrate that periodontal disease contributes not only to incident cardiovascular events but also to long-term mortality, reinforcing the systemic burden of chronic oral inflammation. These findings align with our results and support the hypothesis that tooth loss may represent a cumulative biomarker of prolonged systemic inflammatory exposure.

Emerging evidence highlights the central role of matrix metalloproteinases (MMPs) in linking periodontal disease to cardiac dysfunction [17,18]. MMPs are key enzymes involved in extracellular matrix degradation during periodontal tissue breakdown, and elevated systemic levels have been consistently reported in individuals with periodontitis. Notably, circulating MMP-2 and MMP-9 levels decrease following successful periodontal therapy, indicating that periodontal inflammation contributes directly to systemic MMP burden [18]. MMPs also participate in adverse myocardial remodeling by degrading collagen within the cardiac extracellular matrix, promoting ventricular dilation, fibrosis, and ultimately heart failure [19]. Recent studies further suggest that MMP activation interacts with oxidative stress pathways and may potentiate myocardial injury in inflammatory states [18,20].

Oxidative stress also plays a critical role in this pathway. Cardiovascular diseases are characterized by chronically elevated ROS (reactive oxygen species) levels, and several MMPs are both activated by ROS and capable of amplifying oxidative stress through feedback mechanisms. This bidirectional interaction exacerbates myocardial injury and contributes to left ventricular dysfunction after MI [21]. Because periodontal disease is similarly associated with increased local and systemic oxidative stress, the convergence of MMP activation and ROS dysregulation provides a compelling mechanistic explanation for the observed association between severe tooth loss and heart failure [22].

Beyond periodontal inflammation, tooth loss may reflect cumulative exposure to adverse health factors, including poor nutrition, socioeconomic disadvantage, multimorbidity, and limited access to preventive care [23,24,25]. These broader determinants must be acknowledged when interpreting the observed associations. In this context, tooth loss may serve as a clinically relevant marker of overall health burden and systemic vulnerability rather than a direct causal mechanism [26].

Additionally, tooth loss, through its nutritional consequences, can lead to obesity, dyslipidemia, and hypertension, conditions strongly associated with HF, as reflected in our study, where obesity and hypertension were significantly more prevalent among HF patients [7,27].

### 4.2. Comparison with Previous Studies

Our findings are consistent with previous epidemiological research showing an association between periodontal disease, tooth loss, and increased cardiovascular risk, including coronary heart disease and stroke [3,28,29]. Lee et al. [10] found that tooth loss is associated with an increased risk of MI, HF, ischemic stroke and all-cause mortality. Yan et al. [7] illustrated that moderate to severe periodontitis is associated with an increased risk of heart failure, independent of cardiovascular risk factors. Nonetheless, Kim et al. [30] observed that a higher number of missing teeth was associated with increased mortality risk, underscoring the potential impact of oral health on overall health outcomes. Molinsky et al. [31] remarked the association between periodontal and heart failure as well as unfavorable changes in CRP levels. Similarly, Aminoshariae et al. [32] reported that tooth loss is a significant risk factor for cardiovascular disease and mortality. However, our study adds novel insights by focusing specifically on HF after MI, a complication that greatly impacts prognosis and quality of life. Few prior investigations have directly examined this relationship, making our work an important contribution to the field. Our study is among the first to link tooth loss specifically with post-MI HF outcomes rather than general cardiovascular risk.

Several additional studies reinforce the systemic implications of poor oral health. Recent meta-analyses demonstrate that tooth loss is associated not only with coronary artery disease but also with increased risk of stroke, atrial fibrillation, and cardiovascular mortality [33,34]. Large-scale cohort studies from Finland, Sweden, and Japan similarly report that individuals with severe tooth loss exhibit higher levels of systemic inflammatory markers and greater long-term cardiovascular risk [6,9,32]. These findings align with our observations and emphasize the need to consider oral health as a component of global cardiovascular risk assessment [35,36].

The limited sample size represents a major constraint in interpreting the present findings. While the observed associations between severe tooth loss and post-MI heart failure were statistically significant, they should be regarded as exploratory rather than confirmatory. Larger, multicenter studies are necessary to validate these associations and determine whether tooth loss exerts an independent effect beyond established metabolic and cardiovascular risk factors.

An important consideration is that tooth loss can result from multiple causes, including dental caries, trauma, prosthetic treatment planning, or periodontal disease. Because our study did not collect detailed information on the etiology of missing teeth, we cannot determine the specific contribution of periodontitis to the observed associations. However, epidemiological data indicate that loss of >20 teeth is strongly associated with severe periodontitis in adults, whereas limited tooth loss (< 10 teeth) is less specific and may reflect caries or restorative extraction planning rather than periodontal destruction. This may explain why the strongest association with heart failure in our study was observed in the >20 teeth category, representing the group most likely to have experienced long-standing periodontal inflammation. Future studies should incorporate structured assessments of periodontal history or validated questionnaires addressing periodontal symptoms (loose teeth, bleeding, mobility) to better differentiate causes of tooth loss.

A notable contribution of this work is its focus specifically on post-MI heart failure, an outcome that has received substantially less attention in the oral–cardiovascular literature. Most prior studies have evaluated coronary disease incidence or general cardiovascular mortality, whereas our analysis explores ventricular dysfunction in an acute MI population-an area where data remain limited [6,30,32,33].

It is also important to consider that tooth loss may act as a broader marker of poor general health, frailty, or cumulative inflammatory burden, rather than a direct causal factor. Individuals with extensive tooth loss often share multiple systemic risk characteristics, like lower socioeconomic status, multimorbidity, poorer access to care, and suboptimal lifestyle factors, which may collectively contribute to worse cardiac outcomes. Our results therefore highlight both biological and socioeconomic dimensions of oral–systemic interactions.

### 4.3. Clinical Implications

The data suggest that oral health assessment could be integrated into cardiovascular risk stratification models. Identifying patients with severe tooth loss may help clinicians recognize individuals at higher risk of developing HF after MI. Severe tooth loss reflects not only the cumulative burden of chronic oral disease but also systemic inflammatory activity, altered nutrition, and clustering of traditional cardiovascular risk factors such as obesity and hypertension. Taken together, these elements create a high-risk profile that favors the progression from acute ischemic events to chronic heart failure.

Oral health likely acts synergistically with systemic factors such as diabetes, obesity, dyslipidemia, and hypertension. Accordingly, the relationship between tooth loss and heart failure may reflect convergent inflammatory, metabolic, and vascular mechanisms rather than a direct, independent effect. Future work should compare post-MI heart failure with other post-MI outcomes, such as recurrent ischemia, ventricular arrhythmias, and rehospitalization, to delineate whether tooth loss is a specific predictor of HF or a broader marker of systemic vulnerability.

The implications of this research extend beyond cardiology into preventive medicine and public health. Periodontal treatment and promotion of oral hygiene could represent cost-effective strategies to reduce systemic inflammation and improve long-term cardiac outcomes. While additional longitudinal and interventional studies are needed to confirm causality, the evidence presented here suggests that oral health should no longer be overlooked in the comprehensive management of cardiovascular patients.

### 4.4. Strengths

This study possesses several noteworthy strengths. First, it bridges two traditionally distinct fields- oral and cardiovascular health- by investigating the association between tooth loss and heart failure in patients with prior myocardial infarction, a population in whom data remain limited. Second, the study uses a relatively well-characterized cohort of 200 MI patients with detailed clinical and echocardiographic data, allowing for robust adjustment of confounding variables such as age, sex, diabetes, and smoking. Third, the analysis integrates both clinical and quantitative cardiac parameters (ejection fraction, heart failure status), enhancing internal validity. Fourth, by categorizing tooth loss severity, the study captures a gradient of oral health deterioration, providing a more nuanced understanding of the relationship between oral status and cardiac outcomes. Finally, the work highlights a clinically simple, noninvasive, and readily observable marker—number of missing teeth—that could serve as a useful indicator for identifying post-MI patients at higher risk of heart failure in routine clinical practice.

### 4.5. Limitations

Several limitations of this study should be acknowledged. First, the cross-sectional design precludes causal inference; while an association between tooth loss and heart failure was observed, temporal relationships cannot be established. Second, the sample size, although adequate for exploratory analysis, limits statistical power to detect subtle associations or perform detailed subgroup analyses. Third, the assessment of tooth loss did not include clinical parameters of periodontal disease activity, tooth decay, or prosthetic rehabilitation, which might have provided more comprehensive insight into oral health status. Fourth, the etiology of tooth loss was not recorded. Because not all missing teeth are attributable to periodontitis, misclassification bias is possible, particularly in patients with limited tooth loss. This limitation likely attenuated the associations in groups with fewer missing teeth but does not diminish the relevance of the findings in the > 20 teeth group, which is epidemiologically more representative of severe periodontitis.Fifth, the diagnosis of heart failure was based on echocardiographic criteria and clinical assessment but did not incorporate biomarkers such as NT-proBNP, which might improve diagnostic precision. Sixth, residual confounding cannot be entirely excluded because factors such as socioeconomic status, nutritional patterns, oral hygiene behaviors and healthcare access were not available in the dataset. Lastly, the study population was drawn from a single center, which may limit generalizability to broader or ethnically diverse populations.

### 4.6. Future Directions

Future research should aim to clarify the causal mechanisms linking tooth loss and heart failure following myocardial infarction. Longitudinal cohort studies are needed to determine whether poor oral health precedes or accelerates cardiac dysfunction over time. Incorporating inflammatory biomarkers, periodontal disease indices, and oral microbiome profiling could help elucidate biological pathways connecting oral inflammation to myocardial remodeling. Interventional studies evaluating whether periodontal therapy or oral rehabilitation can improve cardiac outcomes would provide valuable evidence for causality and clinical translation [16,37]. Furthermore, integrating oral health assessments into routine cardiovascular risk stratification may facilitate early identification of high-risk individuals. Multicenter studies with larger, ethnically diverse populations are recommended to confirm generalizability and support the development of multidisciplinary preventive strategies bridging dental and cardiac care.

## 5. Conclusions

In summary, this study demonstrates a strong association between extensive tooth loss and the presence of heart failure among patients with previous myocardial infarction. Although it demonstrates a robust association between tooth loss and post-MI heart failure, its cross-sectional design does not allow causal inference. Longitudinal cohort studies and interventional trials evaluating periodontal treatment are needed to confirm causality and to determine whether improving oral health can actively reduce heart failure risk in post-MI patients.

Tooth loss may serve as a simple clinical indicator of systemic inflammatory burden. It may also represent a marker rather than a direct contributor to adverse cardiac remodeling.

Integrating oral health examination or simple oral-health screening, such as documenting tooth-loss patterns, into cardiovascular care could improve risk stratification after myocardial infarction and support multidisciplinary preventive strategies. Collaboration between cardiology and dental professionals may facilitate earlier identification of high-risk individuals and promote more holistic prevention strategies.

## Figures and Tables

**Table 1:** Overview of Study Design, Population, Variables and Procedures.

Category	Description
Study design	Cross-sectional observational study
Setting	Cardiology Department, County Emergency Clinical Hospital, Oradea (Romania)
Study period	April 2024–September 2025
Participants	200 consecutive adults with angiographically confirmed MI
Inclusion criteria	− Age ≥ 40 years − Acute MI confirmed by coronary angiography − Oral examination completed − Written informed consent
Exclusion criteria	− Active systemic infection or malignancy − Autoimmune/inflammatory diseases (rheumatoid arthritis, lupus) − Recent dental procedures (< 3 months) − Non-ischemic cardiomyopathy, prior heart failure, congenital heart diseases, severe valvular heart diseases − Missing clinical/echocardiographic data
Dental assessment	Standardized tooth count; prosthetic status; oral hygiene questionnaire
Examiner reliability	Cardiologists trained by dentist; Cohen’s kappa = 0.89
Cardiac assessment	Echocardiography using biplane Simpson; coronary angiography
Definition of HF	LVEF < 40% or clinical HF
Confounders included	Age, sex, diabetes, smoking
Statistical analysis	Descriptive statistics; ANOVA; chi-square tests; multivariate logistic regression; ROC analysis

**Table 2:** Distribution of Tooth Loss and Cardiac Parameters.

Tooth Loss Category	Ejection Fraction (mean ± SD)	Heart Failure (%)
0–8	50.1 ± 6.2	22.5
9–20	44.8 ± 6.7	35.0
>20	38.4 ± 5.6	62.5

Note: Values are presented as mean ± standard deviation (SD) or percentages. *p* for trend < 0.001.

**Table 3:** Multivariate logistic regression analysis of heart failure predictors.

Variable	Adjusted_OR	95%_CI	*p*-Value
Tooth loss (>20 vs. 0–8)	2.45	1.15–5.23	0.02
Age (per year)	1.04	1.01–1.08	0.03
Male sex	1.22	0.65–2.28	0.55
Diabetes	1.56	0.80–3.05	0.18
Smoking (current)	1.18	0.62–2.21	0.61

**Table 4:** Sensitivity analysis excluding diabetic patients.

Variable	Adjusted OR	95% CI	*p*-Value
Tooth loss (>20 vs. 0–8)	2.31	1.10–4.89	0.03
Age (per year)	1.03	1.00–1.07	0.04
Male sex	1.10	0.58–2.05	0.65
Smoking (current)	1.15	0.60–2.15	0.59

Model performance: AUC = 0.77; Hosmer–Lemeshow *p* = 0.49.

## Data Availability

The raw data supporting the conclusions of this article will be made available by the authors on request. The data are not publicity available due to privacy reasons.
